# Investigating the misguided path of the superior epigastric artery: an anatomical study of 40 human donors

**DOI:** 10.1007/s12565-025-00884-9

**Published:** 2025-07-29

**Authors:** Edin Ahmic, Paul Swatek, Paul Bamberg, Iurii Mykoliuk, Andrej Roj, Anton Busau, Jörg Lindenmann

**Affiliations:** 1 Department of Thoracic and Hyperbaric Surgery, Medical University Graz, Auenbruggerplatz 29/3, A-8036 Graz, Austria; 2 Center for Anatomy and Cell Biology, Division of Anatomy, Medical University of Vienna, Vienna, Austria

**Keywords:** Larrey`s fissure, Trigonum sternocostale, Superior epigastric artery

## Abstract

This study addresses a longstanding discrepancy between the observed course of the superior epigastric artery (SEA) in human donors and its depiction in many German-language anatomy textbooks. While the SEA typically runs ventral to the diaphragm, several textbooks inaccurately describe it as passing through the sternocostal triangle (Larrey’s fissure). Anatomical dissections were performed on 40 formalin-fixed human donors at the Medical University of Vienna. The thoracic and abdominal walls were systematically dissected, and the course of the SEA was documented. Additionally, historical literature from Dominique Larrey to present-day sources was analyzed to trace the origin and persistence of the inaccurate anatomical description. In all specimens, the SEA remained ventral to the pleural and peritoneal cavities and did not traverse Larrey’s space. The misinterpretation appears to have originated in the 19th century through a chain of misreadings that began with Joseph Hyrtl and was later codified by Friedrich Merkel and was subsequently perpetuated in various German-language anatomical references. This study challenges the traditional textbook depiction of the SEA’s pathway through Larrey’s space and suggests that historical anatomical literature should be revised to reflect accurate topographical relationships.

## Introduction

The central aim of this anatomical study is to clarify the course of the superior epigastric artery (SEA). The starting point is an obvious and unexplained discrepancy between the topographic relations presented in anatomical specimens and the generally represented German-language textbooks’ opinion.

In nature, the SEA is a vessel of the chest and abdominal wall. As one of the terminal branches of the internal thoracic artery, it commences in the angle between the ribs and transversus thoracis muscle. To reach the ventral aspect of the dorsal wall of the rectus sheath, it continues in the same layer and thus enters the anterolateral abdominal wall. The SEA anastomoses with the inferior epigastric artery within the rectus abdominis muscle in a highly variable manner (Loukas et al. 2023; Terfera and Jegtvig 2022).

In contrast to these established facts, some German textbooks suggest that the superior epigastric artery (SEA) passes through the sternocostal triangle (Larrey's fissure) of the diaphragm (Sobotta. Lehrbuch. 2019; Aumüller et al. 2020; Maier and Winkelmann 2024; Urban & Fischer 2020; Waschke and Paulsen 2022; Krenek 2023; Waldeyer 2012; Pschyrembel 2022; Kretz 2021; Bauer and Wolfram 2022; Corts 2023). This gap represents a weak region between the sternal and costal parts of the diaphragm and connects the pleural cavity with the peritoneal cavity. Therefore, embryological as well as topographic anatomical considerations lead to the conclusion that this course has to be considered unrealistic. Our study, carried out on a significant number of anatomic specimens (*N *= 40) has revealed that these textbook views are really misleading.

In addition, by exact study of anatomy books published since the days of Dominique Larrey we have found that unexpectedly Joseph Hyrtl’s (Hyrtl 1846, 1847, 1860) writings may have contributed to the development of the erroneous description. In contrast to his remaining and generally outstanding work he slipped in some misunderstanding of the original description given by Larrey who had definitely stated that the sternocostal triangle is a space "free of any vessels" (Larrey 1831). However, because a SEA passing through Larrey’s fissure could potentially resemble a rare anatomical variation, this description has also been carefully considered to ensure accuracy. We hypothesized that the SEA does not pass through Larrey’s space and that the persistent textbook description is based on historical misinterpretation rather than anatomical variation.

## Materials and methods

This study is based on anatomical specimens from 40 voluntary body donors. The thoracic and abdominal walls were dissected layer by layer, and the course of the SEA was meticulously documented. The focus was exclusively on the anterior part of the thoracoabdominal wall and the anterior insertions of the diaphragm. Only one side (left) was dissected in each specimen to maintain consistency and due to standard procedural constraints in the dissection course setting. Although both internal thoracic arteries were exposed during the dissection by removal of the sternum, only the left side was analyzed and documented for consistency across all specimens.

The study included 40 formalin-fixed human donors (33 male, 7 female), selected consecutively from the available pool during academic dissection courses, specifically the mandatory course "Organ Morphology" at the Medical University of Vienna. Age ranged from 62 to 89 years, based on donation records. Donors voluntarily, and with compensation, provided their bodies to the Center for Anatomy and Cell Biology for educational and research purposes. All procedures were carried out in accordance with institutional ethical guidelines and documented donor consent. The project was approved by the Ethics Committee of the Medical University of Vienna.

All specimens underwent standardized formalin-based perfusion fixation via the femoral artery, followed by long-term immersion in preservative solution. Although formalin fixation may cause slight tissue shrinkage or distortion, we did not observe any distortion in the area of interest, and topographical relationships appeared preserved. Due to the nature of body donation, precise clinical data were not accessible.

## Results

### The anatomic results

The internal thoracic artery (ITA) arises from the subclavian artery as its sole branch from the caudal side. The origin of the ITA is covered by the internal jugular vein. It then descends obliquely to the first rib and is positioned between the subclavian vein (ventrally) and the pleural dome (dorsally). At this level, the phrenic nerve typically runs along its lateral side, crossing it either ventrally or dorsally and then continues parallel to it. The vagus nerve runs approximately 2–3 cm lateral to the ITA. As the artery continues its vertical descent, it reaches the anterior thoracic wall, where it runs parallel to the sternum at a mean distance of 1.1 ± 0.3 cm, aligning itself with the inner surface of the costal cartilages. Thus, it lies dorsally to the costal cartilages and intercostal spaces, and ventrally to the endothoracic fascia and parietal pleura. In each of the upper five or six intercostal spaces, the ITA gives off anterior intercostal arteries to the intercostal spaces. Thin, fine branches called perforating branches (ventral cutaneous branches) emerge from within to reach the skin of the chest wall. The ITA also gives off other important branches, namely the mediastinal branches to the mediastinum, sternal branches to the sternum, and a long branch, the pericardiacophrenic artery, which supplies the muscular parts of the diaphragm.

**Fig 1:**
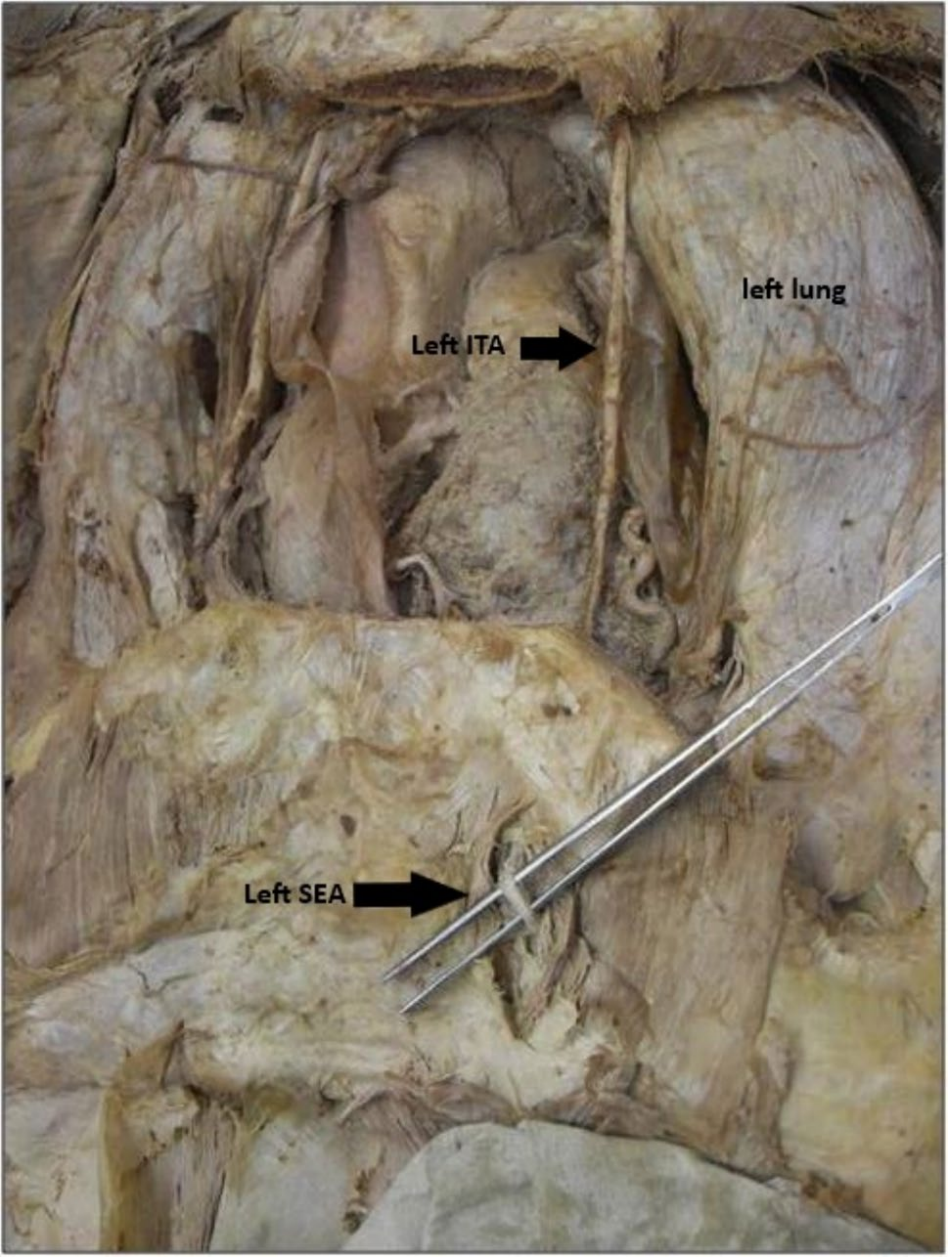
Dissection view of the thoracic region, highlighting the anatomical relationship between the internal thoracic artery (ITA) and the superior epigastric artery (SEA). The ITA is running along the inner surface of the thoracic wall, while the SEA is shown emerging below, likely at the point where it transitions from the thoracic cavity under the ribcage.

**Fig 2:**
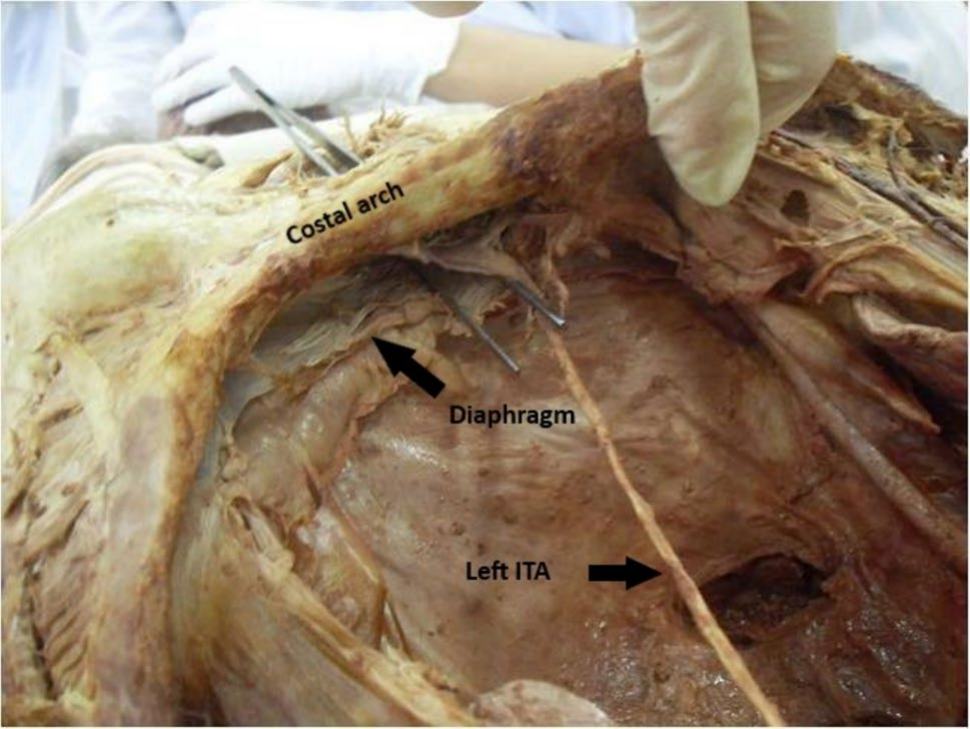
The internal thoracic artery (ITA) is visible running along the inner surface of the thoracic wall, while the superior epigastric artery (SEA) is seen emerging just under the ribcage, transitioning toward the abdominal region

In all 40 cases (*n* = 40 arteries, left side only), the internal thoracic artery bifurcated at the level of the sixth rib in 20 donors and at the seventh rib in the remaining 20 donors into its two terminal branches, the SEA and the musculophrenic artery. The musculophrenic artery runs laterally along the last costal cartilage, travels a variable distance within the muscular layer of the diaphragm, which it supplies, and terminates at the 10th rib. The SEA continues the course of the ITA (Fig. 1). It passes between the costal arch and the transversus thoracis muscle to enter the rectus sheath (Fig. 2). There, it initially runs ventral to the posterior layer of the rectus sheath, which is formed cranially by the transversus thoracis muscle and later by the transversus abdominis muscle. At a variable height, it enters the dorsal surface of the rectus abdominis muscle and anastomoses within this muscle with the inferior epigastric artery.

### Literature review

The first response regarding the misinterpretation of the course of the SEA was published by Kubik and Steiner in 1973 in the *Swiss Journal of the History of Medicine and Sciences.* (Kubik and Steiner 1973) Like in German anatomy textbooks, in the French literature, there are also discrepancies, as highlighted by the study of Baudoin et al. (2003).

Jean-Dominique Larrey (1766–1842), the greatest French military surgeon of his time, published his collected surgical experiences in the work "Surgical Clinic." In the chapter "On Wounds of the Pericardium and the Heart," he describes new approaches to opening the pericardium in cases of effusion (Mihalkovics 1888).

The first anatomist to mention Larrey was Joseph Hyrtl (1811–1894). In his "Textbook of Human Anatomy" from 1846, he writes in the chapter on the diaphragm: "Between the costal segment that originates from the 7th rib cartilage and the one that arises from the xiphoid process, there exists a triangular gap through which the pleura and peritoneum come into contact. Larrey advised puncturing the pericardium through this gap” (Hyrtl, 1846, p.312). (Hyrtl 1846) A year later, in his "Handbook of Topographical Anatomy" (1847), Hyrtl states: “According to Larrey, there is a gap which he recommended for performing pericardial puncture.” (Hyrtl 1847, pp. 399–400) The decision to associate Larrey’s name with this triangular muscle-free zone was made 13 years later, in the 4th edition of Hyrtl’s Topographical Anatomy (Hyrtl 1860). In this edition, a new chapter titled "Larrey's Diaphragmatic Space" was introduced, where it is stated: “Between the bundles of the diaphragm that originate on either side from the xiphoid process and the 7th rib cartilage, there is, according to Larrey, a gap that is covered or closed from below by the peritoneum and from above by the pericardium.” Hyrtl, 1860, p. 541) (Kubik and Steiner 1973).

The first transmission of the name "Larrey's Diaphragmatic Space" for the sternocostal triangle, as coined by Hyrtl, was made by Mihalkovics from Budapest in 1888, an anatomist from the Vienna School (Mihalkovics 1888). He was followed by Merkel (1899) and Joessel-Waldeyer (1899). However, the majority of authors at that time did not even mention Larrey’s name. Then, the situation suddenly changed, and from around 1900, we find the term "Larrey's Space" being used as a synonym for the sternocostal triangle in almost all German-language anatomy textbooks (Kubik and Steiner 1973). Although Larrey was Napoleon’s military surgeon, interestingly, his name does not appear in French anatomical literature in this context until as late as 1930 (Testut and Latarjet 1948; Hovelacque et al. 1937; Paturet 1951). This example illustrates that the content of most books, even today, is still heavily influenced by preceding literature in the same language.

Among French authors, similar disagreements and controversies can be found in anatomical literature, with some also suggesting that the superior epigastric artery passes through Larrey’s diaphragmatic space (Rouvière and Delmas 1992; Bouchet and Cuilleret 1991). In contrast, Netter (in the French version) holds a different view, correctly asserting that the superior epigastric artery runs between the costal arch and the transversus abdominis muscle (Netter 1997; Baudoin et al. 2003).

Unfortunately, Hyrtl’s description does not clarify whether he referred to the sternocostal triangles on both sides of the body under the term "Larrey's space" or only to the left-sided weakness through which Larrey originally described his access to the pericardium. The latter seems likely since Hyrtl described the space as "covered or closed from below by the peritoneum and from above by the pericardium." (Hyrtl 1860, p. 541) In most anatomical textbooks published since then, Larrey’s space is described as the passage for the paired superior epigastric vessels. (Kubik and Steiner 1973)

In 1957, surgeons Zenker and Grill (1957) named the right-sided triangle after Morgagni and the left-sided one after Larrey. They explained that the right diaphragmatic hernia was named after Morgagni in 1761 due to a hernia he found during an autopsy at this site. Thus, it entered the literature as the "Morgagni hernia." Interestingly, Morgagni actually described the occurrence of diaphragmatic hernias in both sternocostal triangles. (Kubik and Steiner 1973; Morgagni 1823).

Regarding the vessels passing through this area, various terms are found in the literature. While Luschka (1863), Merkel (1899), and Joessel-Waldeyer (1899) identified the "Vasa mammaria interna," or the internal thoracic artery (ITA), in this space, more recent textbooks (such as Eisler in 1912, Sieglbauer in 1927, and Schubert in 1964) describe the transition of the internal thoracic artery into the superior epigastric vessels in Larrey’s space (Kubik and Steiner 1973).

From the facts just described, we can conclude that the error regarding “the internal thoracic artery or its abdominal terminal branch, the superior epigastric artery, passing through the sternocostal triangle” does not originate with Larrey. On the contrary, he described during his operation that "no significant vessel is encountered along this entire route." Hyrtl, who first referred to the space as “Larrey's space,” also did not explicitly mention the presence of vessels in this weak spot. The first mistaken connection between these anatomical facts seems to have been made by Luschka in 1863, as he wrote: “The size of these gaps filled with loose, fatty tissue between the sternal and costal parts, through which the internal thoracic artery takes its course...” (Luschka 1863, p. 158). He also referenced Larrey, stating that "on the right side, this triangular space is covered by the pleura, whereas on the left it is free and can therefore be used for pericardial puncture according to Larrey's method." (Kubik and Steiner 1973; Luschka 1863).

In Friedrich Merkel’s textbook from 1899 (Merkel 1899), it is stated unequivocally: "There are two weak spots that are only closed by loose connective tissue: the space between the sternal and costal portions, Larrey’s space, and the space between the costal and vertebral portions. The former abuts the pleura on the right and the pericardium on the left. Through these, the internal thoracic vessels enter the abdominal cavity" (Merkel 1899, p. 339). This interpretation was subsequently adopted by all authors (Kubik and Steiner 1973).

## Discussion

Our results are consistent with the findings of studies by Ludwig (Ludwig 1955), Gurriet, and Thevenat (Guerrier and Thevenat 1955), who, as early as 1955, pointed out the incorrect representation in anatomy textbooks. Interestingly, these publications have so far not been acknowledged by the authors and editors of German-language anatomical works; in all the standard textbooks, the false description of the course of the superior epigastric artery through Larrey’s space persists. These authors (Sobotta. Lehrbuch Anatomie. 2019; Aumüller et al. 2020; Maier and Winkelmann 2024; Urban & Fischer 2020; Waschke and Paulsen 2022; Krenek 2023; Waldeyer 2012; Pschyrembel 2022; Kretz 2021; Bauer and Wolfram 2022; Corts 2023) overlook the fact that, for this to be true, the vessel would have to cross from the thoracic cavity into the abdominal cavity. This would mean that it would run along the anterior surface of the liver on the right side and more or less "freely" in front of the stomach on the left side. Such courses are simply impossible, especially since a separate ventral mesentery would have to develop for this intra-abdominal vessel. This entire description is therefore absurd. The internal thoracic artery and its direct continuation, the superior epigastric artery, are vessels of the chest and abdominal wall that run ventral to the pleural and peritoneal cavities.

In American and Japanese literature (such as in Gray (Williams et al. 1995) and Okajima (1933)), the correct description can sometimes be found. Similarly, in classic anatomical textbooks, which are still largely in Latin (like those of Vesalius (1604) and Verheyen (1981)—the latter also in German, being the first German-language anatomy textbook from 1708), the accurate details are also present. Of particular significance in this comparison is Verheyen’s anatomy textbook, written in its original Latin version (Verheyen 1699). The logical and clear interpretation of the course of the internal thoracic artery and the superior epigastric artery is evident when reading this work from 1699.

Our literature review revealed that Larrey himself had nothing to do with this misconception. Instead, it appears to have originated from Friedrich Merkel’s textbook (Merkel 1899), where—contrary to his usual precise observations—he made an evident translation and interpretation error. Several decades later, anatomists Ludwig (1955), Guerrier, and Thevenat (1955) simultaneously attempted to correct this description in their publications. Unfortunately, few have followed their anatomically accurate observations to this day.

In this anatomical study, we were able to determine that there are generally no gaps or potential openings between the sternal and costal parts of the diaphragm. We have also repeatedly emphasized that Larrey did not penetrate the diaphragm when performing a pericardial puncture. The parts of the diaphragm we have been discussing are not gaps or openings, but rather sections where the diaphragm is formed by connective tissue fibers rather than muscle.

Furthermore, Kubik and Steiner (1973) were able to demonstrate in their study that the misleading description found in anatomical textbooks resulted from a chain of misinterpretations and citations. It was highly interesting to trace this chain of arguments back over more than a hundred years and to observe the often differing interpretations by the same authors at various points and in different editions of their works. Additionally, it is quite surprising that, after nearly five hundred years of the history of scientific human anatomy (since Vesalius' “Fabrica” in 1543) (Vesalius 1604), there are still "gaps" in a field often considered "complete."

## Conclusion

We can conclude that there is no apparent reason for the course of the superior epigastric artery through the so-called Larrey’s space, i.e., the sternocostal triangle of the diaphragm. We did not observe any such anatomical variation in our study, nor are there any publications on this topic. The erroneous description, which has been perpetuated in German-language anatomy textbooks for almost 200 years, can be traced back to a chain of misinterpretations that began with Joseph Hyrtl and were codified and propagated by Friedrich Merkel in 1899. Although Ludwig in 1955 and Kubik and Steiner in 1973 have already pointed this out, little has changed to this day.
